# Perforating Granuloma Annulare Mimicking Psoriasis

**DOI:** 10.7759/cureus.9983

**Published:** 2020-08-24

**Authors:** Joanne S Jacob, Gregory Krenek, Jaime Tschen

**Affiliations:** 1 Dermatology, Baylor College of Medicine, Houston, USA; 2 Dermatology, Conroe Dermatology Associates, Conroe, USA; 3 Dermatology, St. Joseph Dermatopathology, Houston, USA

**Keywords:** perforating granuloma annulare, pga, dermatopathology

## Abstract

Perforating granuloma annulare (PGA) is a rare inflammatory condition characterized by transepithelial elimination of necrobiotic collagen with granulomas in the dermis. It commonly presents as umbilicated papules or pustules on the extremities and dorsal hands. The distribution of PGA can be described as generalized or localized, with only 9% of patients presenting with a single lesion. Herein, we report an unusual presentation of PGA as a single localized plaque on the forearm that resembled psoriasis.

## Introduction

Granuloma annulare (GA) is a benign, granulomatous condition that presents with annular papules on the hands and feet [1]. One rare subtype of GA is perforating granuloma annulare (PGA), characterized by histopathologic evidence of granulomas surrounding trans-epidermal elimination of necrotic material [2]. PGA has a typical clinical appearance of umbilicated papules or pustules with crusting [2-3]. We report an unusual plaque psoriasis-like presentation of localized PGA in a 67-year-old male without systemic symptoms.

## Case presentation

A 67-year-old man presented with a tender plaque on the dorsal left forearm that gradually progressed in size over a one-month period. The patient had not previously been treated with any topical or systemic treatments. Past dermatologic history included multiple actinic keratoses on the scalp and back treated with cryotherapy and topical 5-fluorouracil. His other medical history includes for hypertension and well-controlled type II diabetes mellitus.

**Figure 1:**
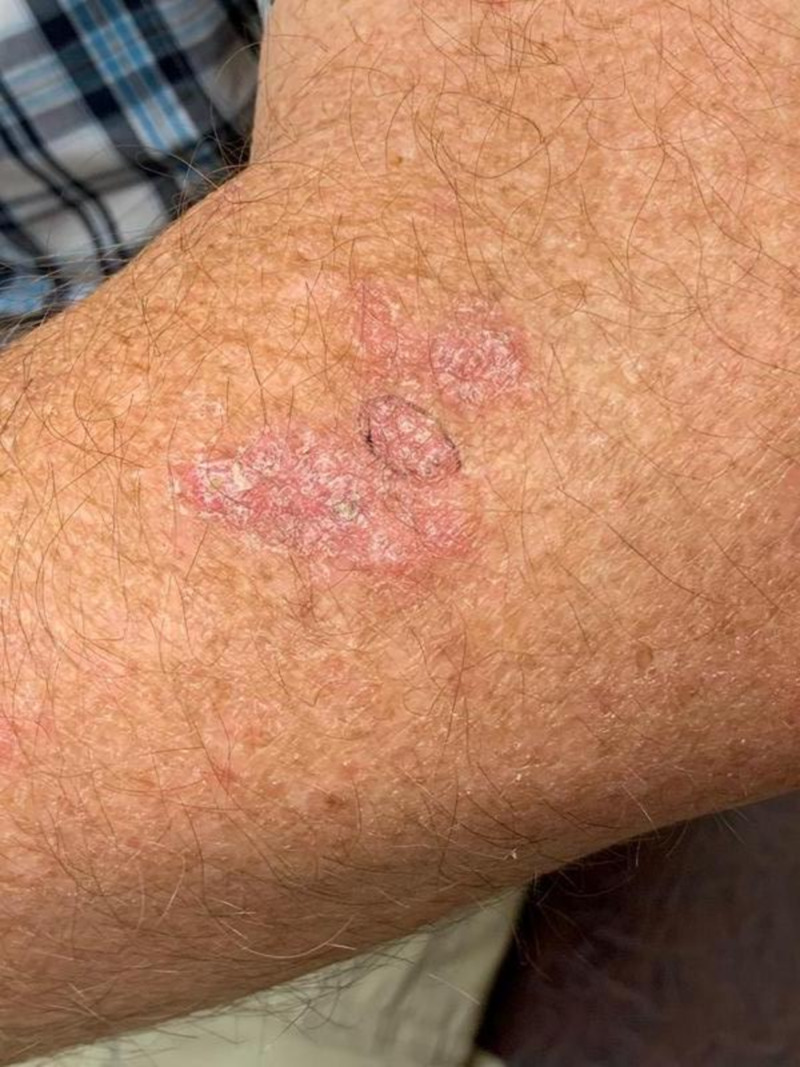
Image taken at follow-up appointment 2.5 x 4 cm asymmetric pink plaque with overlying scaling and crusting. Image was taken before biopsy. Area to be biopsied was marked with the black circle.

Physical exam revealed a single asymmetric pink tender plaque with overlying scaling and crusting on the left forearm. Other exam findings included scattered seborrheic keratoses on the trunk. The initial clinical suspicion was between inflammatory dermatoses, such as atopic dermatitis or contact dermatitis, and psoriasis. The patient was first treated with 0.1% triamcinolone ointment. After one month, repeat examination revealed progression of the tender pink plaque, which was measured to be approximately 2.5 x 4 cm (Figure 1). A shave biopsy of the skin measuring 0.8 x 0.8 cm was performed at the center of the plaque. The patient was sent home after biopsy with a prescription for combination 0.01%/0.045% halobetasol propionate and tazarotene topical therapy.

**Figure 2:**
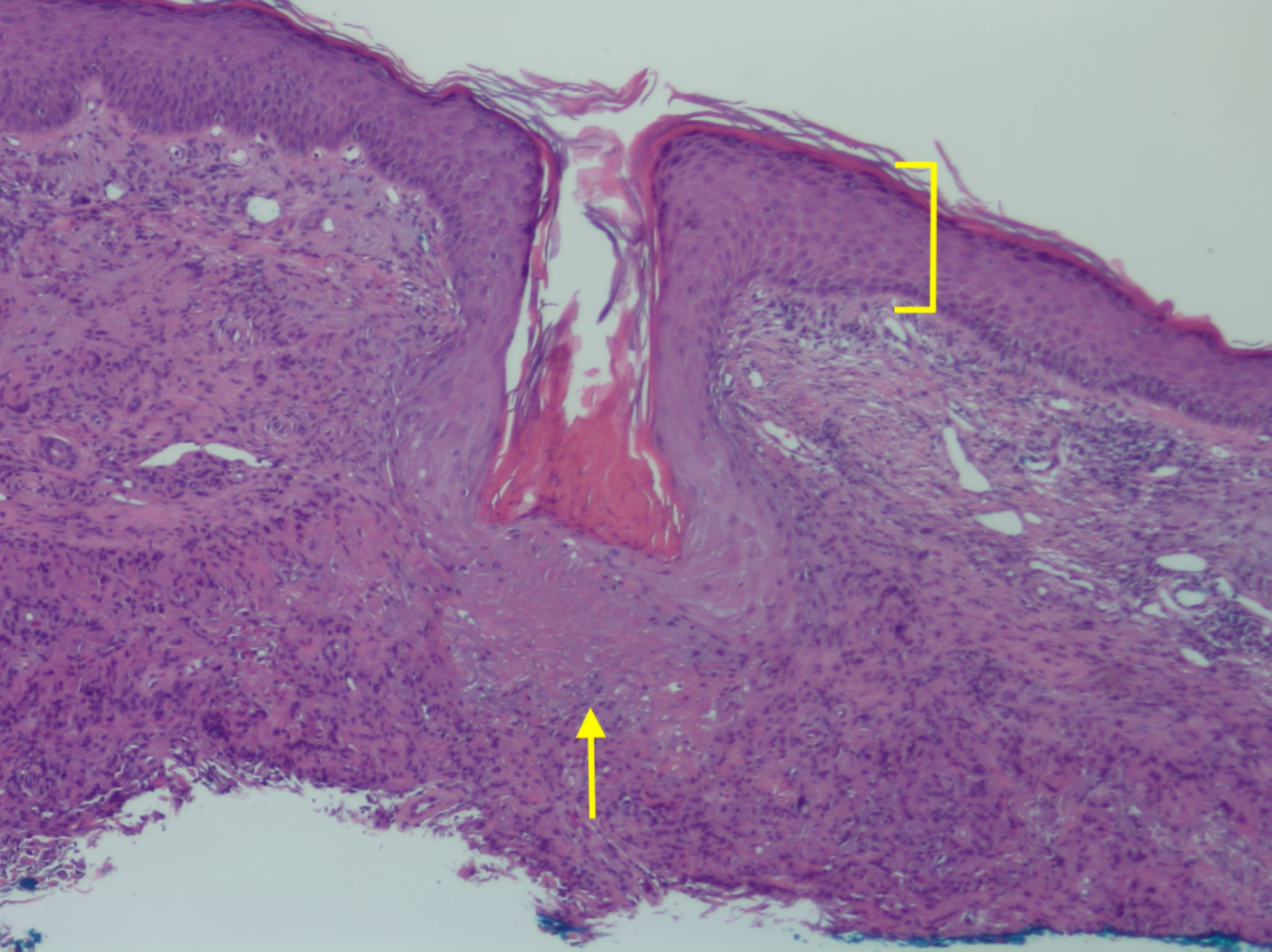
Transepidermal elimination Bracket demonstrates pseudoepitheliomatous hyperplasia of epidermis and arrow points to trans-epidermal elimination of granulomas. Hematoxylin and eosin stain with 100x magnification.

**Figure 3:**
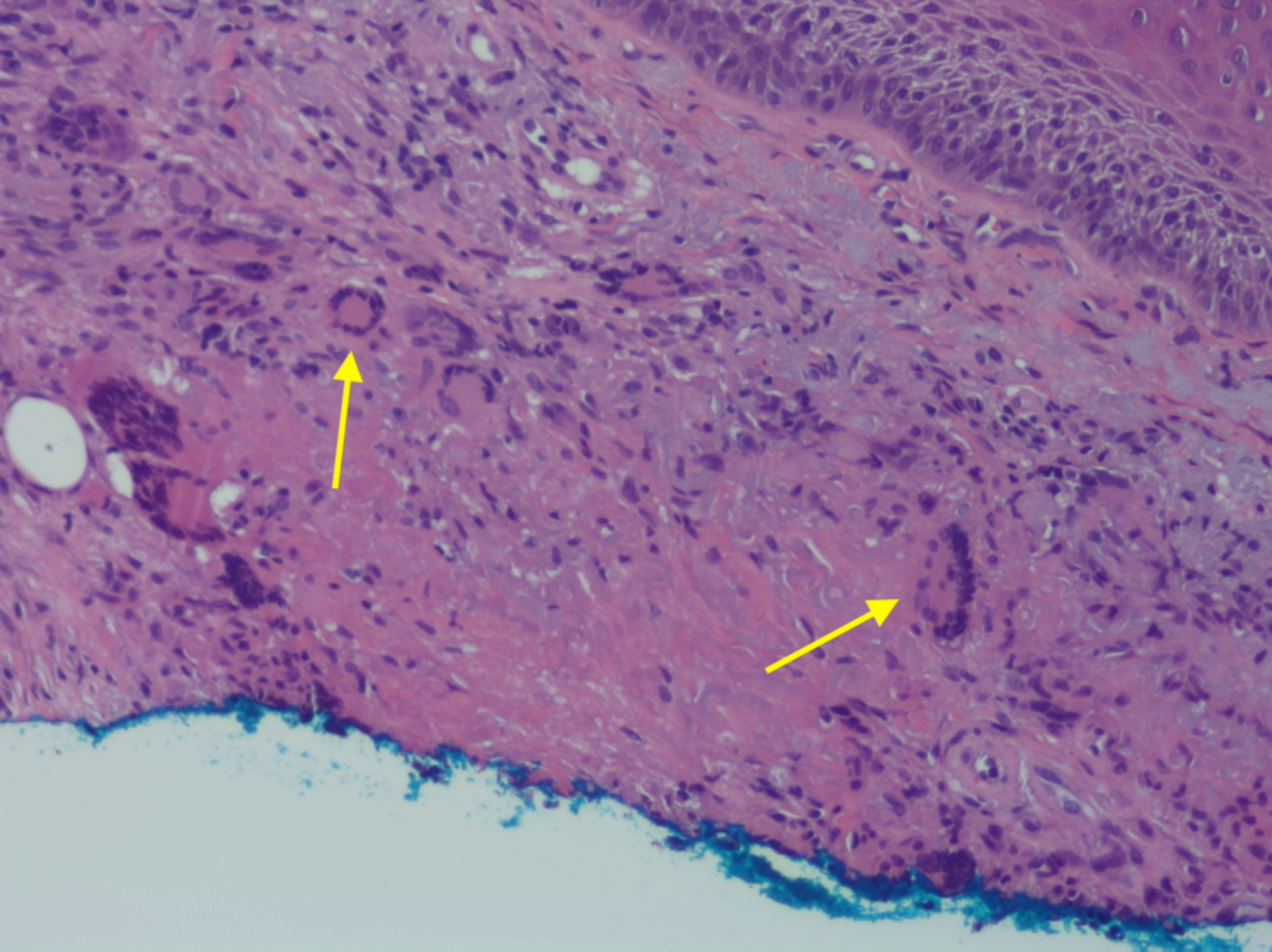
Evidence of granulomas Granulomatous inflammation with multinuclear giant cells in the superficial and mid-dermis. Necrobiosis is seen at the tails of the arrows. Hematoxylin and eosin stain with 200x magnification.

**Figure 4:**
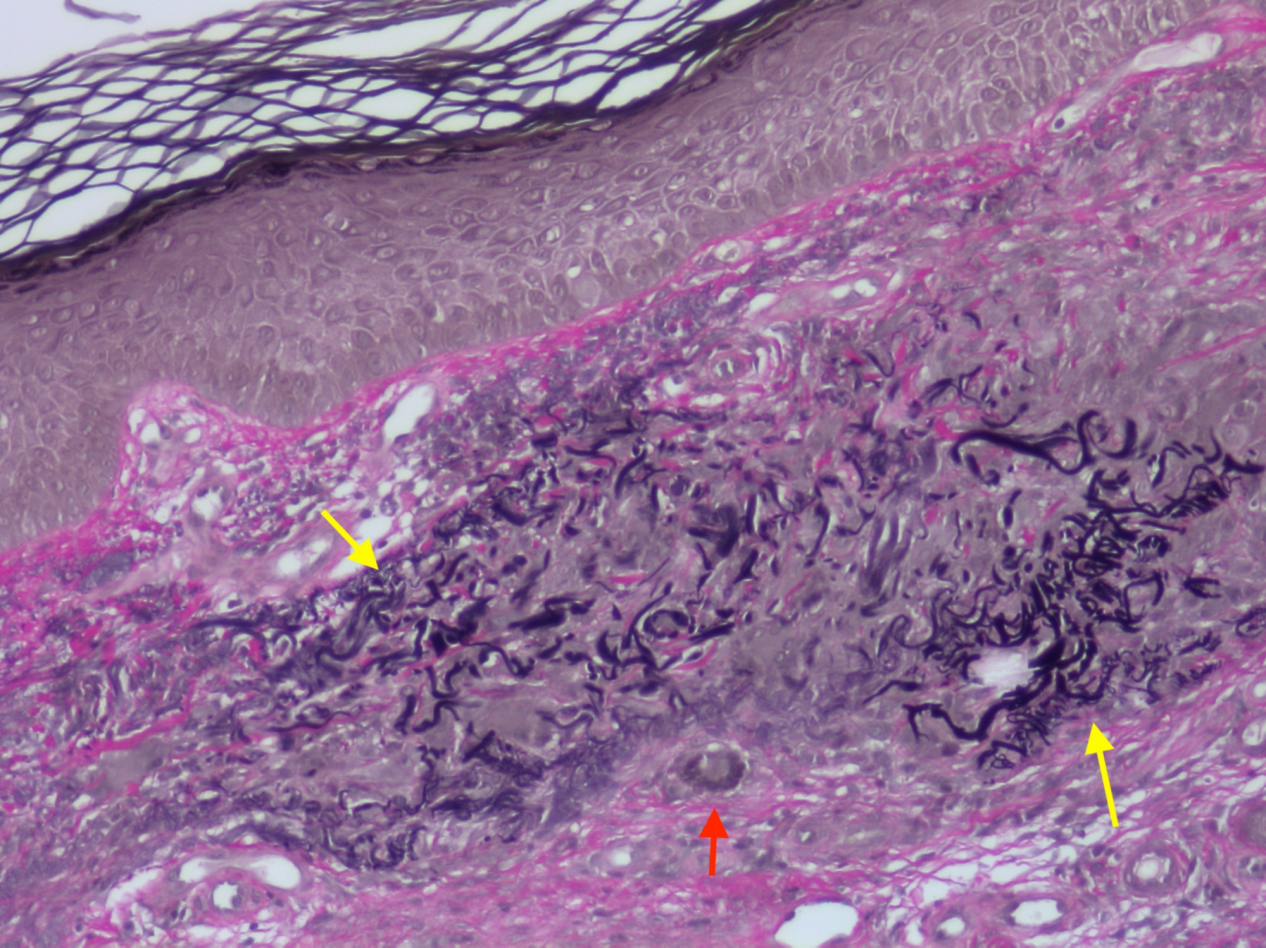
Elastic fibers within granuloma Damaged elastic fibers (yellow arrows) are seen in the granuloma. A giant cell (red arrow) is seen at the periphery without phagocytized elastic fibers. Verhoeff-Van Gieson (VVG) stain with 100x magnification.

**Figure 5:**
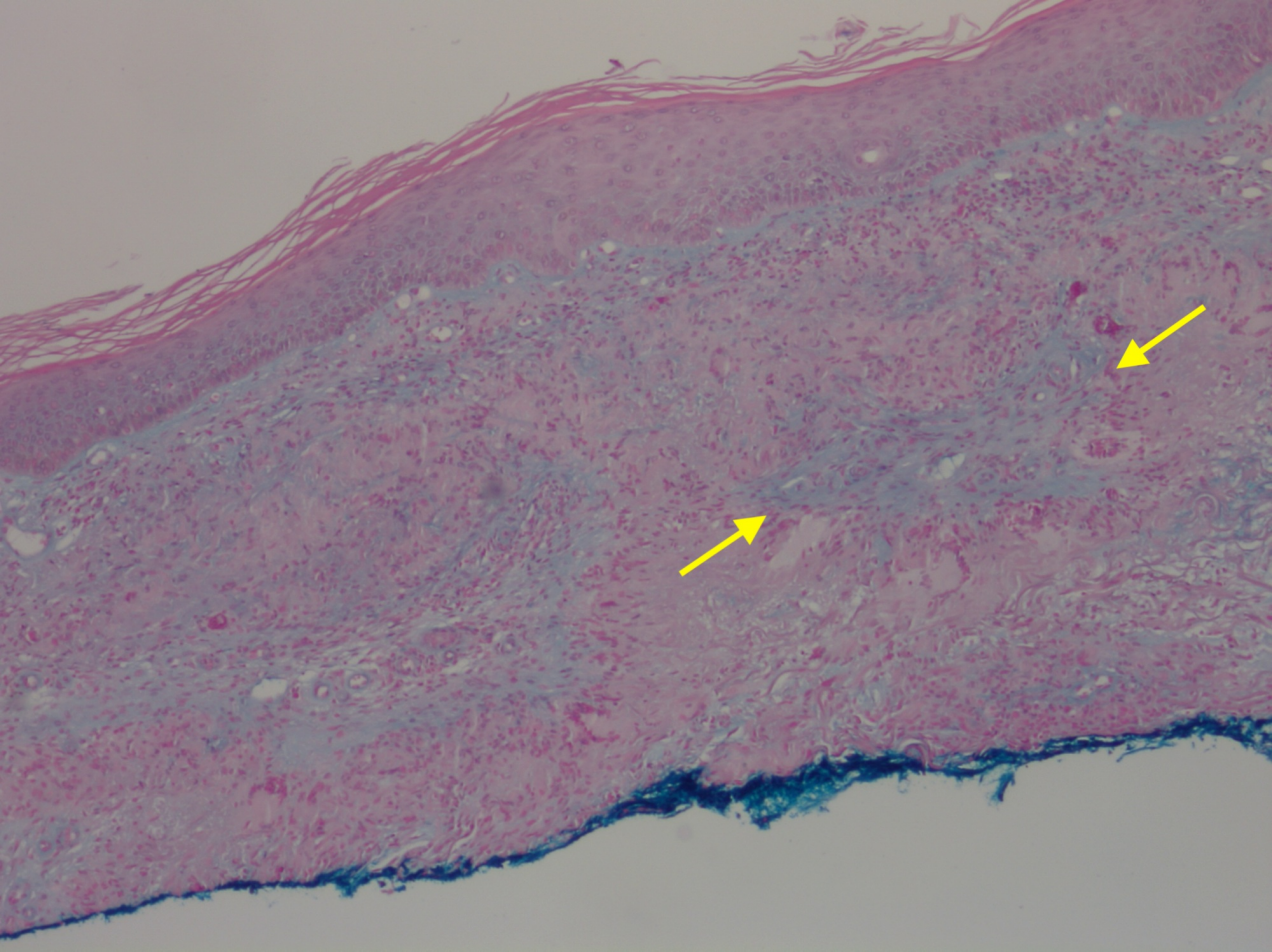
Mucin within granuloma Abundant mucin is seen in the center of the granulomas in areas of necrobiosis (yellow arrows). Alcian Blue stain with 100x magnification.

Histopathologic examination of the shave biopsy demonstrated pseudoepitheliomatous hyperplasia of the epidermis and trans-epidermal elimination of granulomas (Figure 2). Granulomatous inflammation with multinuclear giant cells and occasional damaged elastic fibers were present in the superficial and mid-dermis (Figure 3). Periodic acid-Schiff, Gram, and acid-fast bacilli stains were negative. Stains for elastin and mucin were also conducted, demonstrating elastic fibers and mucin within the granulomas (Figures 4-5).

Overall, the histopathologic features were consistent with a diagnosis of PGA. The patient was made aware of the diagnosis one week after the visit and treatment regimen of halobetasol propionate and tazarotene ointment was continued while waiting for a follow-up appointment. Follow-up was delayed due to stay-at-home orders and had not been yet conducted at the time of article publication.

## Discussion

PGA was first described in 1971 in two patients with lesions on the hands. Histopathology demonstrated perforation of the epidermis by necrotic material and dermis granulomas, and the new term PGA was coined [4]. Since then, it is recognized as a rare subtype of granuloma annulare. Overall pathogenesis of granuloma annulare is thought to be due to a helper T-cell based delayed hypersensitivity reaction to exogenous factors such as minor trauma and ultraviolet radiation [2].

Histopathologic features of PGA include granulomas with multinuclear histiocytes arranged in a diffuse or palisading pattern around a trans-epidermal protrusion of necrotic collagen [2]. The differential diagnosis of PGA most often includes other conditions in which organic material is protruded through the epidermis, such as reactive perforating collagensosis, reactive perforating dermatosis, perforating folliculitis, elastosis perforans serpiginosa, and perforating calcific elastosis [2,5]. PGA is primarily distinguished from other perforating conditions by the presence of granulomas in the dermis. Granulomas may not be visible on every section of tissue prepared for slides, making it difficult to distinguish from other perforating conditions if not examined in serial sections [4]. The presence of granulomas can lead to suspicion of mycobacterial infection or tuberculoid papule but can be ruled out by a negative acid-fast Bacilli stain as well as tissue biopsy for culture.

PGA is classically described as flesh to red-colored umbilicated papules or pustules ranging from 1-5 mm. These papules often have an overlying scale, crusting, or exudate, and are often arranged in an annular form [2]. The distribution of PGA can be described as generalized or localized, with only 9% of patients presenting with a single lesion [6]. To our knowledge, only one other case report has demonstrated a plaque psoriasis-like presentation [7]. In that case, multiple progressive painful plaques presented on the hands and extremities and were accompanied by systemic polyarthralgia, myopathy, and dactylitis. Notably, the localized PGA case we are presenting only had a single large plaque. Our patient lacked systemic involvement and had a more indolent presentation, suggesting that similar lesions may be underreported if a biopsy is not performed. 

Treatment options for PGA includes complete excision, psoralen, and ultraviolet A therapy, intralesional triamcinolone, and high-dose corticosteroids [6,7]. Spontaneous resolution was seen in 77% of patients in one study [6]. Case reports have demonstrated improvement with topical tacrolimus in cases of steroid-resistant PGA [8-9]. Inhibition of interleukin (IL)-2 and tumor necrosis factor (TNF)-a by tacrolimus is thought to reduce granulomatous inflammation in PGAs [8]. Other studies have demonstrated improvement with topical vitamin E or oral vitamin D therapy at varying doses [1]. Overall, treatment for PGA demonstrates variable efficacy and are based on case reports and case series. It is challenging to perform clinical studies due to the rarity of the condition.

## Conclusions

PGA is a rare skin condition that can uncommonly present as a single painful psoriasis-like plaque. In our patient, the diagnosis was made by histopathologic examination after the lesion failed to respond to empiric anti-inflammatory therapy. Clinicians should be aware of this unusual presentation of localized PGA and have a low threshold for biopsy when refractory to conventional therapy.
